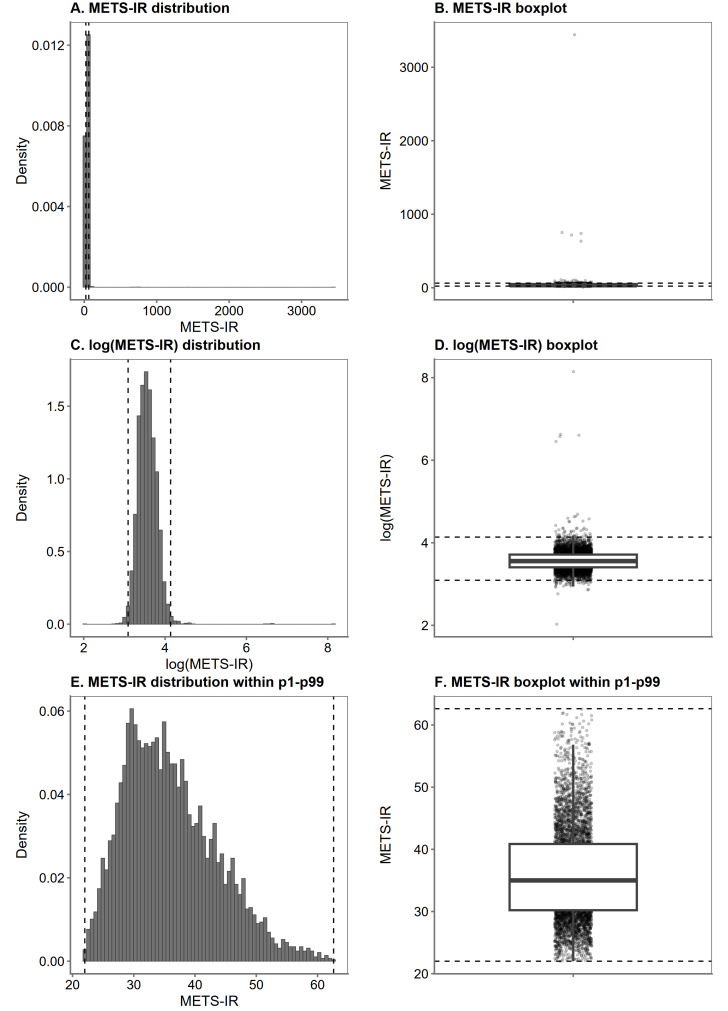# Substitutional value of METS-IR for biochemical components of life’s essential 8 in predicting incident mild cognitive impairment: A longitudinal cohort study

**DOI:** 10.1097/MD.0000000000049278

**Published:** 2026-06-12

**Authors:** Xuzhou Zhu, Chenyue Wang, Qinghua Cao, Qian Xu, Xiaoli Guo, Chunbo Liu

**Affiliations:** a School of Biological and Behavioural Sciences, Queen Mary University of London, London, UK; b The Queen Mary School, Jiangxi Medical College, Nanchang University, Nanchang, Jiangxi, China; c Department of Neurology, The First Affiliated Hospital of Ningbo University, Ningbo, Zhejiang, China; d Department of Anesthesia Operating Room, The First Affiliated Hospital of Ningbo University, Ningbo, Zhejiang, China; e Department of Nursing, The First Affiliated Hospital of Ningbo University, Ningbo, Zhejiang, China.

**Keywords:** life’s essential 8, machine-learning, METS-IR, mild cognitive impairment, sarcopenia

## Abstract

Insulin resistance is closely associated with mild cognitive impairment (MCI) and sarcopenia. The predictive utility of the metabolic score for insulin resistance (METS-IR) for MCI across different sarcopenia strata and its potential as a substitute for life’s essential 8 (LE-8) remains unclear. This prospective cohort study used data from the China Health and Retirement Longitudinal Study. Participants aged ≥45 years without baseline MCI or memory-related disease and with biomarker and cognitive data were included. METS-IR was calculated from fasting blood glucose, triglycerides, high-density lipoprotein cholesterol, and body mass index. LE-8 was scored using an adapted American Heart Association framework. Associations between METS-IR and incident MCI stratified by sarcopenia status were assessed using Cox proportional hazards models and restricted cubic splines. Machine-learning analyses using 11 classifiers and stratified 10-fold cross-validation evaluated the incremental and substitutional value of METS-IR within LE-8-based prediction models. A total of 4980 participants were included. Higher METS-IR was associated with a less favorable cardiometabolic profile. In multivariable-adjusted Cox models, compared with the lowest quartile of METS-IR, hazard ratios for incident MCI were 0.94 (95% confidence interval [CI]: 0.79–1.11) for quartile 2, 0.65 (95% CI: 0.53–0.78) for quartile 3, and 0.72 (95% CI: 0.59–0.87) for quartile 4. Restricted cubic spline analyses showed a significant nonlinear association between METS-IR and incident MCI in the overall sample (*P* for overall < .001, *P* for nonlinear = .017). A significant overall association was also observed in the possible sarcopenia subgroup, although nonlinearity was not statistically significant. No significant nonlinear or overall association was observed in the nonsarcopenia or confirmed sarcopenia subgroups. In prediction analyses, LE-8-based models achieved moderate discrimination (area under curve, AUC: 0.525–0.696). Adding METS-IR yielded a modest but statistically significant improvement only in AdaBoost (ΔAUC = +0.00487, *P* < .05). When selected biochemical LE-8 components were substituted by METS-IR, discrimination improved in several algorithms in multiple models, particularly in multilayer perceptron (*P* < .01). METS-IR showed a significant nonlinear association with incident MCI, with the lowest risk observed at intermediate-to-higher METS-IR levels. The incremental and substitutional value beyond LE-8 was limited, model-dependent, and requires external validation.

## 1. Introduction

Global population aging is placing an escalating strain on healthcare systems worldwide.^[1]^ Within this demographic shift, age-related functional and cognitive decline has emerged as a critical public health challenge. Cognitive impairment in the elderly is conceptualized as a continuous spectrum, encompassing subjective cognitive decline, mild cognitive impairment (MCI), and Alzheimer disease.^[2]^ MCI is frequently regarded as a prodromal phase of neurodegenerative diseases. It specifically refers to a transitional clinical state characterized by objective and subjective evidence of cognitive decline, while activities of daily living remain largely intact, and the individual does not meet the diagnostic criteria for dementia.^[3]^ Research on cognitive function in the elderly indicates that the prevalence of MCI among individuals aged 60 and over in China is approximately 15.5%. Without intervention, the average annual conversion rate to Alzheimer disease is 20%.^[4,5]^ Notably, approximately 24% of elderly patients with MCI may revert to a cognitively normal status in the future,^[6]^ suggesting the early diagnosis and prevention of MCI is of paramount significance for reducing the global prevalence of dementia. Sarcopenia is a syndrome characterized by the progressive attrition of skeletal muscle mass, strength, and physical function,^[7,8]^ which is commonly considered a comorbidity of MCI. Mechanistically, sarcopenia and insulin resistance (IR) may synergistically accelerate cognitive decline, as muscle loss impairs glucose disposal, which in turn exacerbates systemic inflammation and oxidative stress in the brain.^[9]^

The American Heart Association’s life’s essential 8 (LE-8) provides a comprehensive framework of cardiovascular health by integrating behavioral and biochemical domains, but it can be data-intensive and does not explicitly capture IR as an integrated pathophysiologic state.^[10]^ Beyond its role in cardiovascular health, recent studies have suggested a potential association between higher LE-8 scores and better cognitive outcomes, though the mechanisms remain to be fully elucidated.^[11]^ In contrast, the metabolic score for insulin resistance (METS-IR) is a pragmatic, noninsulin-based index derived from routinely available clinical measures, and recent evidence links higher METS-IR to poorer cognitive outcomes.^[12]^ Insulin resistance is highly prevalent worldwide, with a recent meta-analysis estimating a pooled global prevalence of 26.53%^[13]^; in China, the age-standardized prevalence of insulin resistance among adults aged ≥25 years has been reported as 29.22%.^[14]^ Despite the mechanistic plausibility that IR contributes to cognitive decline^[15]^ and evidence linking cardiometabolic health metrics to sarcopenia,^[16]^ to our knowledge, no study has yet evaluated whether METS-IR may substitute for LE-8’s metabolic subcomponents without compromising model performance. In this prospective cohort study, we examined the associations between METS-IR and incident MCI across sarcopenia strata. Furthermore, LE-8-based prediction models were utilized to assess the incremental and substitutional value of METS-IR beyond LE-8.

## 2. Methods

### 2.1. Study population

The China Health and Retirement Longitudinal Study (CHARLS) is a nationally representative longitudinal survey of Chinese adults. The national baseline survey was conducted between June 2011 and March 2012 using a stratified, multistage probability sampling design with probability proportional to size. Sampling covered 150 counties/districts, 450 villages/urban communities, across 28 provinces, and targeted households with members aged ≥45 years.^[17]^

We used the Harmonized CHARLS Version D respondent-level dataset, which contains 25,586 unique individuals who participated in at least 1 wave (2011/2013/2015/2018).^[18]^ Based on Harmonized CHARLS D, we additionally merged Wave 1 (2011) information from the household income, health status and functioning, and blood datasets, and cognitive follow-up information collected in Wave 5 (2020).

The harmonized dataset included 25,586 individuals. We excluded: age <45 years (n = 602), unavailable METS-IR components (n = 16,437), missing baseline cognition (2011) (n = 1903), baseline cognition below the threshold and classified as MCI (n = 1230), missing baseline age (n = 29), baseline age not classifiable into age groups (n = 2), baseline doctor-diagnosed memory-related disease (n = 64), baseline medication for memory-related disease (n = 3), and no cognitive follow-up information (n = 336). The final analytic sample included 4980 participants (Table S1, Supplemental Digital Content 1). To assess potential selection bias due to participant exclusions, we further compared baseline characteristics between included and excluded participants (Table S2, Supplemental Digital Content 2).

The original CHARLS study was approved by the Ethical Review Committee of Peking University (IRB00001052-11015 and IRB00001052-11014). All procedures were carried out in accordance with the Declaration of Helsinki, and written informed consent was obtained from all participants prior to their participation.

### 2.2. Biomarker and covariates

#### 2.2.1. Blood collection and laboratory assays

Venous blood was collected by trained Chinese Center for Disease Control and Prevention staff using a standardized protocol. Respondents were asked to fast overnight; blood was collected even if not fasting, and fasting status was recorded in the blood dataset. In this study, we excluded blood glucose data collected in the nonfasting state and did not use these data to calculate METS-IR. Three tubes were collected (complete blood count tube, plasma/buffy coat tube, and glycated hemoglobin A1c [HbA1c] tube), processed under temperature control, and stored frozen before assay at Capital Medical University. Blood-based assays included glucose and lipid measures relevant to this study. HbA1c was measured by boronated affinity HPLC. Lipid and glucose were measured by enzymatic colorimetric tests.

#### 2.2.2. Blood pressure and anthropometrics

Blood pressure was measured using an Omron HEM-7200 Monitor. Three readings were taken with ~45 seconds between measurements. Systolic/diastolic blood pressure summaries were computed as the average of the 2nd and 3rd readings (or 1st and 2nd if the 3rd is unavailable). Height was measured with a Seca 213 stadiometer and weight with an Omron HN-286 scale. Body mass index (BMI) was calculated as weight (kg)/height (m^2^).

#### 2.2.3. Covariates

Diabetes, hypertension, and memory-related diseases were based on doctors’ diagnoses. Age, sex, education level, residence, food-related spending, physical activity, alcohol and nicotine exposure, nightly sleep duration, and medication use were obtained from respondents’ self-reports.

***METS-IR***

METS-IR was calculated as:


$$METS\text{-}IR=\frac{\ln\left[\left(2\times FBG\right)+TG\right]\times BMI}{\ln\;HDL\text{-}C}$$

where FBG is fasting blood glucose, TG is triglycerides, HDL-C is high-density lipoprotein cholesterol, and BMI is body mass index.

### 2.3. Definition of life’s essential 8

We applied the American Heart Association (AHA) LE-8 framework using the original 0 to 100 scoring approach for each metric.^[10]^

CHARLS does not provide a direct dietary structure score aligned with AHA. Therefore, we constructed a diet proxy (diet index) based on household consumption structure, defined as the proportion of “food expenditure” over total food-related spending:


$$\begin{array}{c} Diet\;index=\frac{Food\;expenditure}{Food\;expenditure+Eating\;out+Alcohol/tobacco\;} \end{array}$$

These components correspond to GE006 (food expenditure), GE007 (eating out), and GE008 (alcohol/tobacco).

For the remaining metrics (physical activity, nicotine exposure, sleep, BMI, blood lipids, blood glucose, blood pressure), we applied AHA-consistent cut points and converted each into 0 to 100 scores.

### 2.4. Measurement of cognitive function

Cognitive function was assessed using the CHARLS cognitive battery, which is based on tasks from the Telephone Interview for Cognitive Status. The assessment covered 4 domains: orientation, computation, memory, and drawing. Orientation was evaluated using date-naming items of 4 components (year, month, day of month, and day of week). Computation was measured using serial subtraction (subtracting 7 from 100 five consecutive times). Memory was assessed by a 10-word list test, including immediate recall and delayed recall. Drawing was evaluated by copying overlapping pentagons. A global cognition score was calculated as the sum of the 4 domain scores (orientation 0–4, computation 0–5, memory 0–20, drawing 0–1), with a total range of 0 to 30.^[19]^

Because there is no single universally accepted operational definition of MCI in population surveys, we applied an aging-associated cognitive decline approach, defining MCI as performance at least 1 standard deviation (SD) below the age-specific mean.^[20,21]^ Specifically, baseline age was grouped into 5-year age groups, and within each group we calculated the mean and SD of the global cognition score. Participants with cognition scores < (mean − 1 SD) within their age stratum were classified as having MCI. Baseline MCI cases were excluded, and incident MCI during follow-up was treated as the outcome event.^[22]^

### 2.5. Assessment of sarcopenia status

Sarcopenia was assessed according to the Asian Working Group for Sarcopenia 2019 (AWGS2019) framework, integrating muscle strength, muscle mass, and physical performance.^[23]^ Handgrip strength was measured by trained staff using a Yuejian WL-1000 dynamometer. 2 trials were performed on each hand with the elbow flexed at approximately 90°, and the maximum value for each hand was retained. When both hands were available, we averaged the maxima of the 2 hands to obtain the final grip strength; when only 1 hand was available, the maximum from the available hand was used.^[22]^ Low muscle strength was defined using AWGS2019 cut points: <28 kg for men and <18 kg for women.^[24]^

Physical performance was evaluated using gait speed, the 5-time chair stand test, and the Short Physical Performance Battery (SPPB). Gait speed was measured using a 2.5-meter usual-pace walking test with recorded completion time (there and back), and the chair stand test recorded the time required to rise from a 47-cm chair 5 times with arms folded across the chest. The SPPB additionally incorporated 3 standing balance positions (side-by-side, semitandem, and tandem), each held for 10 seconds, producing a composite score from 0 to 12. Low physical performance was defined as meeting any of the following AWGS2019 thresholds: gait speed <1.0 m/s (equivalently, >2.5 seconds for 2.5 m), 5-time chair stand time ≥12 seconds, or SPPB score < 9.^[22]^

Muscle mass was estimated using an anthropometry-based appendicular skeletal muscle mass equation (ASM):


$$\begin{array}{c} \begin{array}{cc} & ASM=0.193\times weight\left(kg\right)+0.107\times height\left(cm\right)\\ & -4.157\times gender-0.037\times age\left(years\right)-2.631 \end{array} \end{array}$$

where gender is coded as male = 1 and female = 0.^[24]^ We then calculated height-adjusted muscle mass (ASM/height^2^). Low muscle mass was defined as the sex-specific lowest 20th percentile of ASM/height^2^ in the study population, with < 4.89 kg/m^2^ in women and <6.79 kg/m^2^ in men.^[22,25]^

Confirmed sarcopenia was defined when low muscle mass was present in addition to low muscle strength and/or low physical performance. Participants not classified as having confirmed sarcopenia were classified as having possible sarcopenia if they had low muscle strength and/or low physical performance.^[22]^

### 2.6. Statistical analysis

Baseline characteristics were summarized across quartiles of METS-IR (Q1–Q4). Continuous variables were presented as mean ± standard deviation and compared using the Kruskal–Wallis test due to nonnormal distributions, while categorical variables were presented as number (percentage) and compared using the χ^2^ test or Fisher exact test.

For the longitudinal outcome, we constructed time-to-event data using repeated MCI assessments across follow-up waves (2013/2015/2018/2020). Incident MCI was defined as the 1st wave in which MCI was identified, and follow-up time (years) was calculated as the difference between the event wave year and baseline year (2011). Participants without incident MCI were censored at the last wave with a valid cognitive assessment and no MCI. Participants with missing follow-up time or follow-up time ≤0 were excluded from survival analyses. Associations between METS-IR and incident MCI were examined using Cox proportional hazards models. The proportional hazards assumption was formally verified using Schoenfeld residuals, and no violations were detected (all *P* > .05). METS-IR was analyzed as quartiles (Q1 as reference), and tests for linear trend were performed by modeling quartile as an ordinal variable. We reported hazard ratios (HRs) with 95% confidence intervals (CIs) for an unadjusted model (Model 1) and a multivariable model (Model 2) adjusted for age, sex, education, physical activity, sleep, smoking, alcohol use, systolic blood pressure, diabetes, and lipid-lowering medication use. Missing values in the Model 2 adjustment set were handled using multiple imputation by chained equations (MICE), imputing only prespecified covariates (exposure and survival outcomes were not imputed). To evaluate potential nonlinear dose–response relationships, we fitted restricted cubic spline models for continuous METS-IR using the rms framework. For visualization, spline curves were plotted after truncating METS-IR to the 2.5th–97.5th percentile range, using the median METS-IR as the reference. Stratified analyses were conducted to assess effect patterns across sarcopenia status. Cox models were repeated within subgroups defined by baseline sarcopenia status (nonsarcopenia, possible sarcopenia, confirmed sarcopenia).

Machine-learning analyses were implemented to evaluate prediction of incident MCI under internal validation. We prespecified multiple feature sets, including conventional predictors (age, sex, and education) and LE-8 component-score predictors; sets augmented with METS-IR; and progressively reduced sets retaining METS-IR. We trained 11 classifiers: logistic regression, decision tree (DT), support vector machine (SVM), random forest, adaptive boosting (AdaBoost [AB]), extreme gradient boosting (XGBoost [XGB]), light gradient boosting machine (LightGBM [LGBM]), multilayer perceptron (MLP), k-nearest neighbors (KNN), Naïve Bayes, categorical boosting (CatBoost [CB]), and evaluated performance using stratified 10-fold cross-validation with a fixed random seed. The use of 11 algorithms was intended to compare simpler, more interpretable models with more flexible nonlinear models and to assess whether the predictive value of METS-IR was consistent across different model architectures. Missing predictors were handled using multivariable iterative imputation by chained equations (sklearn IterativeImputer). Categorical variables were imputed using the most frequent category. Preprocessing steps (multivariable imputation, standardization of continuous predictors, and 1-hot encoding of categorical predictors) were fit within each training fold and applied to the corresponding validation fold to prevent data leakage. Hyperparameters were tuned using randomized search and then reused within the outer cross-validation procedure for each model-feature-set combination. Out-of-fold predicted probabilities were aggregated to compute area under the receiver operating characteristic curve with 95% CIs via 2000 bootstrap resamples. Model calibration was further assessed using calibration plots and calibration metrics calculated from out-of-fold predicted probabilities, including the Brier score, calibration intercept, calibration slope, and Hosmer–Lemeshow test. Differences in area under the receiver operating characteristic curve between selected feature sets were tested using the DeLong method for correlated receiver operating characteristic curves.^[26]^

For Cox regression analyses, sensitivity analyses evaluated robustness to alternative operational definitions and analytic choices, including redefining MCI using alternative thresholds of −0.8 SD and −1.2 SD below the age-specific mean; a stricter baseline exclusion strategy based on a −1.2 SD cutoff while retaining the main −1 SD definition for incident MCI; repeating Cox models without MICE imputation (complete-case adjustment); and excluding METS-IR outliers by restricting the analysis to participants within the 1st to 99th percentile of METS-IR.

For prediction analyses, the incremental and substitutional evaluations were also repeated after restricting the sample to the 1st to 99th percentile of METS-IR, using the same internal-validation framework.

All analyses were conducted using R 4.5.2 (R Foundation for Statistical Computing, Vienna, Austria) and Python 3.11.9 (Python Software Foundation, Wilmington), with *P* value <.05 considered significant.

## 3. Results

### 3.1. Baseline characteristics

After exclusions, a total of 4980 participants were included in the final analysis. Participants were categorized into quartiles (Q1–Q4) based on the baseline distribution of the METS-IR: the lowest quartile (Q1: 27.11 ± 2.45), 2nd quartile (Q2: 32.52 ± 1.41), 3rd quartile (Q3: 37.74 ± 1.73), and highest quartile (Q4: 52.54 ± 103.50).

The participants with higher METS-IR values tended to be younger, were more likely to be female, and more frequently resided in urban communities. They also exhibited elevated levels of BMI, nonhigh-density lipoprotein cholesterol, fasting blood glucose, HbA1c, and blood pressure, alongside a higher prevalence of diagnosed diabetes, hypertension, and stroke. Notably, the composition of sarcopenia status differed significantly; the high METS-IR group showed a lower proportion of confirmed sarcopenia but a higher prevalence of possible sarcopenia. The distribution across individual components was inconsistent: within the behavioral dimension, scores for diet, nicotine exposure and sleep were higher, whereas physical activity scores were lower. Furthermore, metabolic-related component scores (BMI, blood lipids, blood glucose, and blood pressure) were overall lower in the higher METS-IR quartiles (Table 1). In summary, individuals with elevated baseline METS-IR presented a more unfavorable cardiometabolic health profile. Compared with excluded participants, the included analytic sample differed in several baseline characteristics, particularly education level and sarcopenia status, whereas most cardiometabolic variables showed relatively small standardized mean differences (Table S2, Supplemental Digital Content 2).

**Table 1:** Baseline characteristics stratified by METS-IR quartiles.

Characteristic	Q1	Q2	Q3	Q4	*P* value
METS-IR	**27.11 ± 2.45**	**32.52 ± 1.41**	**37.74 ± 1.73**	**52.54 ± 103.50**	**–**
Age (yr)	59.65 ± 8.94	57.77 ± 8.51	57.52 ± 8.16	56.71 ± 7.82	**<.001**
Sex, n (%)					**<.001**
Female	467 (37.5%)	530 (42.6%)	637 (51.2%)	664 (53.3%)	
Male	778 (62.5%)	715 (57.4%)	608 (48.8%)	581 (46.7%)	
Education, n (%)					**<.001**
Below primary school	480 (38.6%)	395 (31.7%)	389 (31.2%)	373 (30.0%)	
High school and above	134 (10.8%)	173 (13.9%)	225 (18.1%)	197 (15.8%)	
Middle school	298 (23.9%)	334 (26.8%)	311 (25.0%)	367 (29.5%)	
Primary school	333 (26.7%)	343 (27.6%)	320 (25.7%)	308 (24.7%)	
Residence, n (%)					
Rural village	1071 (86.0%)	989 (79.4%)	887 (71.2%)	894 (71.8%)	**<.001**
Urban community	174 (14.0%)	256 (20.6%)	358 (28.8%)	351 (28.2%)	
Alcohol, n (%)					**<.001**
None	702 (56.4%)	755 (60.6%)	810 (65.1%)	864 (69.4%)	
Yes	543 (43.6%)	490 (39.4%)	435 (34.9%)	381 (30.6%)	
Body mass index	19.89 ± 1.79	22.45 ± 1.55	24.74 ± 1.83	31.65 ± 73.46	**<.001**
nHDL-C (mg/dL)	127.19 ± 33.15	138.93 ± 35.60	148.19 ± 36.24	161.29 ± 39.97	**<.001**
FBG (mg/dL)	100.12 ± 18.68	104.30 ± 21.70	109.30 ± 30.21	126.84 ± 54.10	**<.001**
HbA1c (%)	5.11 ± 0.51	5.17 ± 0.62	5.26 ± 0.71	5.60 ± 1.15	**<.001**
SBP (mm Hg)	123.81 ± 20.13	125.59 ± 19.61	129.90 ± 20.16	134.29 ± 20.66	**<.001**
DBP (mm Hg)	71.78 ± 11.42	73.69 ± 11.68	76.78 ± 11.60	79.43 ± 11.99	**<.001**
Diabetes mellitus diagnosed					**<.001**
No	1222 (98.7%)	1189 (96.3%)	1137 (92.7%)	1051 (85.0%)	
Yes	16 (1.3%)	46 (3.7%)	90 (7.3%)	185 (15.0%)	
Hypertension diagnosed					**<.001**
No	1089 (87.8%)	985 (79.5%)	872 (70.4%)	711 (57.2%)	
Yes	152 (12.2%)	254 (20.5%)	366 (29.6%)	531 (42.8%)	
Stroke diagnosed					
No	1228 (98.7%)	1219 (98.3%)	1213 (97.8%)	1208 (97.2%)	**.041**
Yes	16 (1.3%)	21 (1.7%)	27 (2.2%)	35 (2.8%)	
Sarcopenia status					**<.001**
Confirmed sarcopenia	172 (13.8%)	8 (0.6%)	1 (0.1%)	0 (0.0%)	
Possible sarcopenia	537 (43.1%)	621 (49.9%)	607 (48.8%)	618 (49.6%)	
Nonsarcopenia	536 (43.1%)	616 (49.5%)	637 (51.2%)	627 (50.4%)	
LE-8 (score 0–100)					
Diet	48.42 ± 40.99	51.26 ± 42.08	54.46 ± 42.42	57.48 ± 42.93	**<.001**
Physical activity	72.32 ± 44.03	69.13 ± 45.58	68.17 ± 45.83	58.49 ± 48.64	**<.001**
Nicotine exposure	48.73 ± 45.58	55.44 ± 44.74	64.16 ± 41.87	66.54 ± 40.08	**<.001**
Sleep	68.90 ± 33.08	70.05 ± 32.33	72.54 ± 30.88	73.04 ± 31.60	**.006**
Body mass index	99.98 ± 0.85	98.39 ± 6.77	86.51 ± 15.30	64.79 ± 22.38	**<.001**
Blood lipids	78.46 ± 26.21	69.59 ± 28.87	61.94 ± 29.44	51.21 ± 29.46	**<.001**
Blood glucose	96.98 ± 12.26	94.89 ± 16.10	91.77 ± 19.96	83.10 ± 27.74	**<.001**
Blood pressure	69.92 ± 33.75	65.93 ± 34.84	56.89 ± 35.73	47.21 ± 35.70	**<.001**

Categorical variables are shown as n (%); continuous variables are shown as mean ± SD.

Alcohol indicates whether the individual consumed alcohol during the past year. Smoking indicates whether the individual had ever smoked during their lifetime. Diabetes mellitus, hypertension and stroke status were based on doctor’s diagnosis. Sarcopenia status was classified in this study. Baseline characteristics were not imputed, so the summed comorbidity counts do not always equal the total number of participants.

Bold *P* values indicate statistical significance.

DBP = diastolic blood pressure, FBG = fasting blood glucose, HbA1c = glycated hemoglobin A1c, LE-8 = life’s essential 8, METS-IR = metabolic score for insulin resistance, nHDL-C = nonhigh-density lipoprotein cholesterol, Q = quartile, SBP = systolic blood pressure.

### 3.2. Overall and sarcopenia-stratified associations between METS-IR and incident MCI

**Figure 1.**
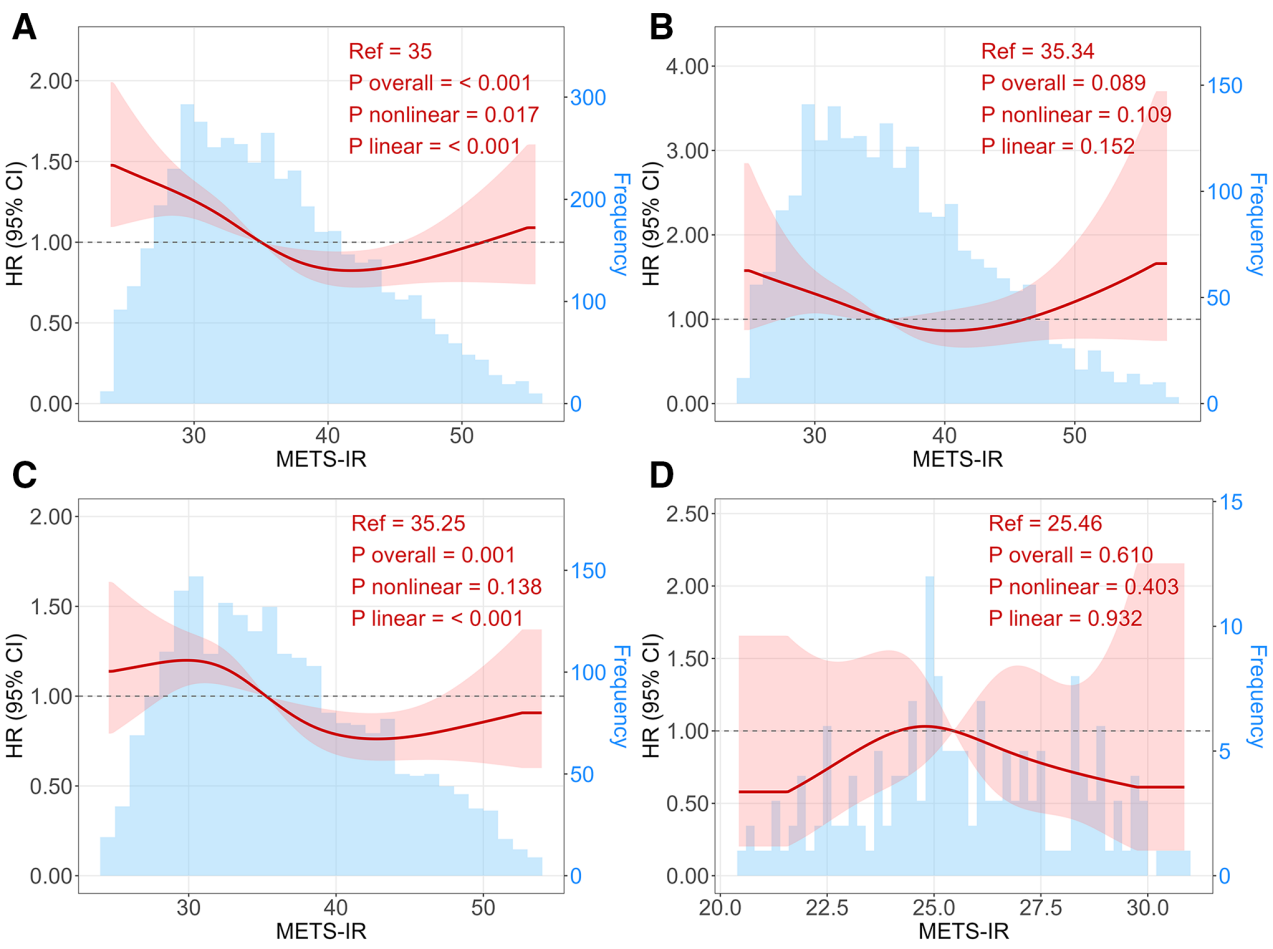
Multivariable-adjusted hazard ratios for MCI based on restricted cubic splines among overall and sarcopenia strata individuals with median METS-IR as reference. Restricted cubic spline curves from Cox proportional hazards models showing hazard ratios (HRs) and 95% confidence intervals (CIs) for incident mild cognitive impairment across continuous metabolic score for insulin resistance (METS-IR). The reference was set at the median METS-IR within each panel (Ref). The solid red line indicates adjusted HRs and the shaded area indicates 95% CIs; the dashed horizontal line denotes HR = 1. The blue histogram (right *y*-axis) shows the distribution (frequency) of METS-IR. Models were adjusted for age, sex, education, physical activity, sleep, smoking, alcohol use, systolic blood pressure, diabetes, and lipid-lowering medication use. Panels: (A) overall sample; (B) nonsarcopenia; (C) possible sarcopenia; (D) confirmed sarcopenia.

Within median follow-up of 9 years, after multivariable adjustment, using Q1 as the reference group, the HRs for the Q2, Q3, and Q4 groups were 0.94 (95% CI: 0.79–1.11), 0.65 (95% CI: 0.53–0.78), and 0.72 (95% CI: 0.59–0.87), respectively in the overall population (Table 2). In the overall population, restricted cubic spline analysis demonstrated a significant overall association between METS-IR and the risk of MCI (*P* for overall <.001), with evidence of nonlinearity (*P* for nonlinear <.05) (Fig. 1A). We further examined the association between METS-IR and incident MCI stratified by sarcopenia status. In the nonsarcopenia subgroup, Q2 and Q3 (vs Q1) were associated with a lower hazard of incident MCI, whereas Q4 was not statistically significant. In the possible sarcopenia subgroup, Q3 and Q4 (vs Q1) were associated with a lower hazard of incident MCI. In the nonsarcopenia population, compared with Q1, the multivariable-adjusted HRs were HR = 0.69 (95% CI: 0.48–0.97) for Q2, HR = 0.56 (95% CI: 0.38–0.80) for Q3, and HR = 0.76 (95% CI: 0.53–1.10) for Q4, with Q3 showing the lowest hazard ratio. In the possible sarcopenia population, compared with Q1, the multivariable-adjusted HRs were HR = 1.01 (95% CI: 0.82–1.23) for Q2, HR = 0.68 (95% CI: 0.54–0.85) for Q3, and HR = 0.71 (95% CI: 0.56–0.90) for Q4, with Q3 showing the lowest hazard ratio (Table S3, Supplemental Digital Content 3). Restricted cubic spline curves suggested that the lowest risk of incident MCI occurred at intermediate METS-IR levels in the overall sample and the possible sarcopenia subgroup. In the nonsarcopenia subgroup, the nonlinear pattern appeared less robust (Fig. 1B and C). However, no consistent dose-response association was observed among participants with confirmed sarcopenia. Restricted cubic spline analysis further showed no significant overall association in the confirmed sarcopenia subgroup (*P* for overall = .610, Fig. 1D).

**Table 2:** Associations of METS-IR and mild cognitive impairment.

Quartile (range)	Number of cases/total	Unadjusted HR (95% CI)	Multivariate model HR (95% CI)	*P* value
Overall (n = 4980)				
Q1 (7.59–30.12)	368/1245	Ref	Ref	–
Q2 (30.12–35.00)	332/1245	0.85 (0.73, 0.99)	0.94 (0.79, 1.11)	.454
Q3 (35.00–41.00)	265/1245	0.66 (0.57, 0.78)	0.65 (0.53, 0.78)	**<.001**
Q4 (41.01–3442.56)	265/1245	0.66 (0.56, 0.77)	0.72 (0.59, 0.87)	**.001**

The multivariate Cox model was adjusted for age, sex, education, physical activity, sleep, smoking, alcohol use, systolic blood pressure, diabetes, and lipid-lowering medication use.

Bold *P* values indicate statistical significance.

CI = confidence interval, HR = hazard ratio, METS-IR = metabolic score for insulin resistance, Ref = reference.

### 3.3. Incremental value of METS-IR in the LE-8 prediction model

**Figure 2.**
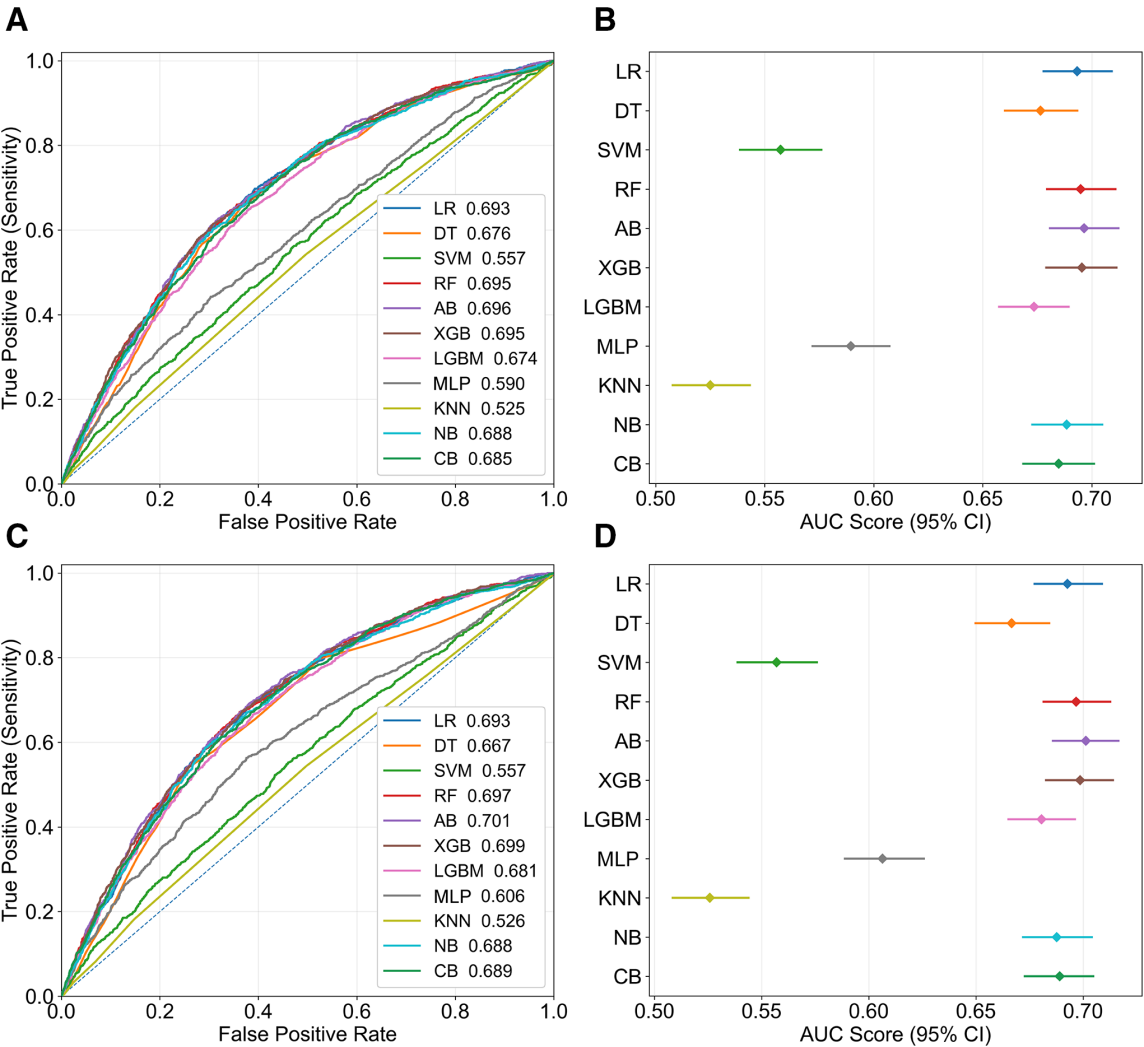
Incremental value of METS-IR beyond life’s essential 8 (LE-8) in predicting incident MCI using machine-learning models. Receiver operating characteristic (ROC) curves and AUC (area under the curve) summaries based on out-of-fold (OOF) predictions from stratified 10-fold cross-validation. (A, B) Life’s Essential 8 (LE-8) component-score predictors; (C, D) LE-8 predictors plus metabolic score for insulin resistance (METS-IR) (incremental model). (A, C) ROC curves for 11 classifiers: logistic regression (LR), decision tree (DT), support vector machine (SVM), random forest (RF), AdaBoost (AB), XGBoost (XGB), LightGBM (LGBM), multilayer perceptron (MLP), k-nearest neighbors (KNN), Naïve Bayes (NB), and CatBoost (CB). The diagonal line represents chance-level discrimination. (B, D) Forest plots showing AUC (95% confidence interval, CI), with 95% CIs obtained via bootstrap resampling of OOF predictions.

Receiver operating characteristic curve analyses using stratified 10-fold cross-validation demonstrated that the LE-8-based prediction model achieved moderate discriminative performance across multiple machine-learning algorithms, with area under the curve (AUC) values ranging from 0.525 to 0.696 (Fig. 2A and B). Among the evaluated models, AB (AUC = 0.696), random forest (AUC = 0.695), and XGB (AUC = 0.695) showed the highest baseline discrimination.

After incorporating METS-IR, predictive performance improved across several algorithms. The highest AUC was observed for AB (AUC = 0.701), followed by XGB (AUC = 0.699) and random forest (AUC = 0.697) (Fig. 2C and D). Compared with the baseline LE-8 model, significant increases in AUC were observed for AB (ΔAUC = +0.00487, *P* < .05), whereas the remaining algorithms did not demonstrate statistically significant improvement (*P* > .05; Table S4, Supplemental Digital Content 4).

### 3.4. Substitutional value of METS-IR in the LE-8 prediction model

In addition to incremental analyses, stepwise substitution analyses were conducted to evaluate the performance of METS-IR–containing reduced-feature models in which selected biochemical LE-8 components were omitted under an identical modeling framework.

**Figure 3.**
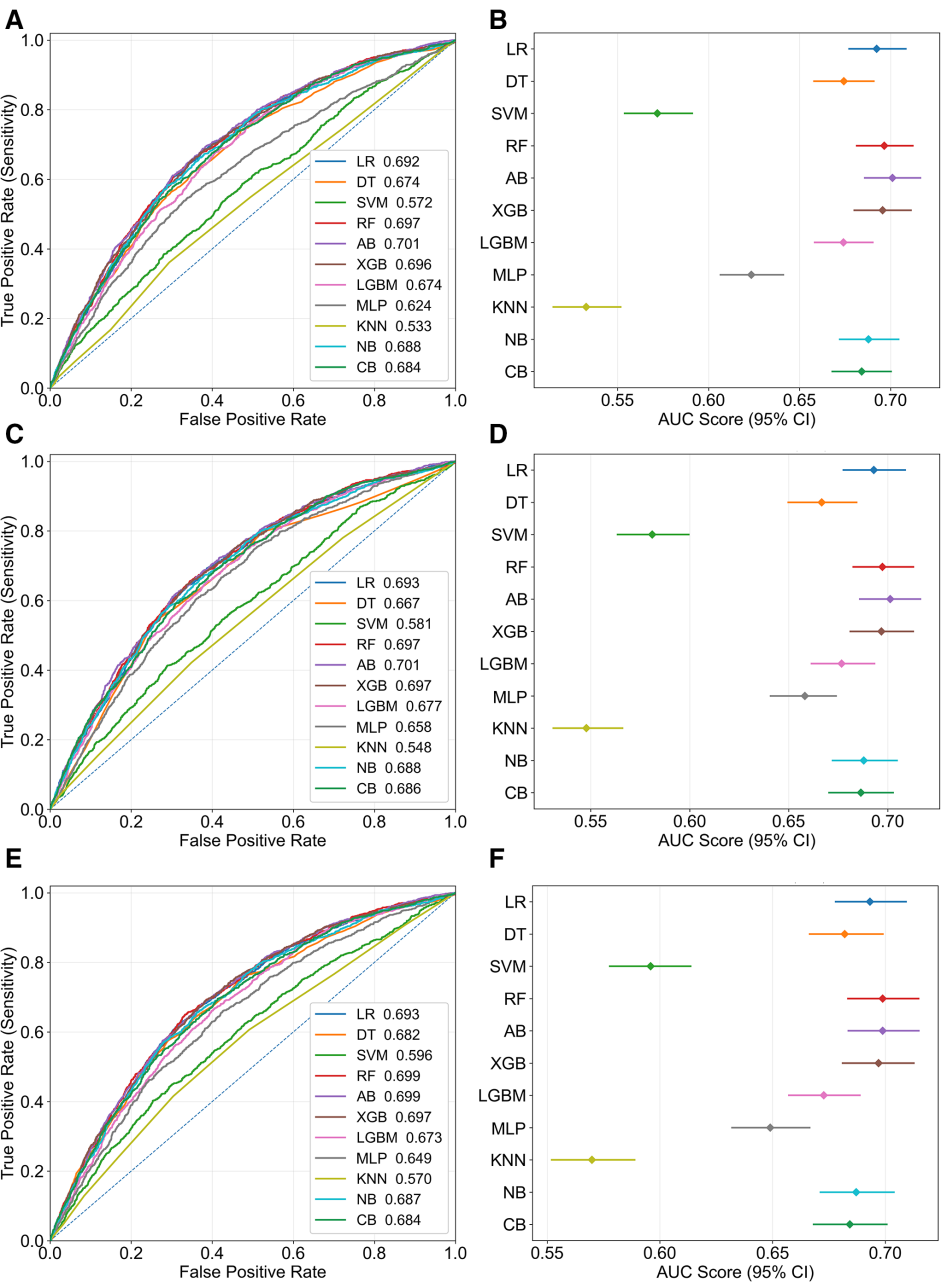
Substitutional value of METS-IR for biochemical components of life’s essential 8 (LE-8) in predicting incident MCI using machine-learning models. Receiver operating characteristic (ROC) curves and AUC (area under the curve) summaries based on out-of-fold (OOF) predictions from stratified 10-fold cross-validation under stepwise substitution feature sets. (A, B) The 2-component substitution model in which metabolic score for insulin resistance (METS-IR) replaces the life’s essential 8 (LE-8) body mass index and blood glucose components; (C, D) the 3-component substitution model in which METS-IR replaces LE-8 body mass index, blood glucose, and blood lipids components; (E, F) 4-component substitution model in which METS-IR replaces LE-8 body mass index, blood glucose, blood lipids, and blood pressure components. (A, C, E) ROC curves for 11 classifiers: logistic regression (LR), decision tree (DT), support vector machine (SVM), random forest (RF), AdaBoost (AB), XGBoost (XGB), LightGBM (LGBM), multilayer perceptron (MLP), k-nearest neighbors (KNN), Naïve Bayes (NB), and CatBoost (CB). (B, D, F) Forest plots showing AUC (95% confidence interval, CI), with 95% CIs obtained via bootstrap resampling of OOF predictions.

In the 2-component substitution models, where BMI and blood glucose were substituted with METS-IR, SVM showed a significant enhancement (ΔAUC = +0.01489, *P* < .001). However, 3 other models showed significant decreases: XGB (ΔAUC = −0.00298), LGBM (ΔAUC = −0.00649), and CB (ΔAUC = −0.00490) (all *P* < .05). The ΔAUC values for these 3 models remained below 0.01 (Fig. 3A and B; Table S5, Supplemental Digital Content 5).

With broader replacement of metabolic components, discrimination improved further. In the 3-component substitution model (minus BMI, blood glucose, and blood lipids), the discriminative performance of 3 algorithms improved significantly: KNN and SVM demonstrated notable improvements, with ΔAUC values reaching 0.02203 (*P* < .05) and 0.02410 (*P* < .001), respectively. The most substantial gain was observed in the MLP model, which reached an AUC of 0.658 (ΔAUC = +0.05169, *P* < .001) (Fig. 3C and D; Table S5, Supplemental Digital Content 5).

Likewise, in the 4-component substitution model (minus BMI, blood glucose, blood lipids, and blood pressure), the most pronounced improvement was observed in KNN, where AUC increased by 0.04408 (*P* < .001), indicating the largest improvement among the evaluated algorithms in discrimination. Comparable increases were identified in MLP (ΔAUC = +0.04252, *P* < .001) and SVM (ΔAUC = +0.03893, *P* < .01), while DT also demonstrated a statistically significant but comparatively smaller gain (ΔAUC = +0.01534, *P* < .001) (Fig. 3E and F; Table S5, Supplemental Digital Content 5). Calibration analyses showed heterogeneous performance across algorithms and feature sets. Across all evaluated models, Brier scores ranged from 0.180 to 0.250. SVM showed relatively favorable calibration in the LE-8 model, incremental model, and 4-component substitution model, whereas most other models showed evidence of imperfect calibration based on the calibration metrics and Hosmer-Lemeshow test (Fig. S1, Supplemental Digital Content 6 and Table S6, Supplemental Digital Content 7).

### 3.5. Sensitivity testing

In Cox regression analyses, the overall and possible sarcopenia findings remained broadly consistent across the 5 sensitivity analyses. In the overall sample, the association between METS-IR and incident MCI remained statistically significant after using alternative MCI thresholds, applying a stricter baseline exclusion strategy, repeating Cox models without MICE imputation, and restricting the analysis to participants within the 1st to 99th percentile of METS-IR. Similar robustness was observed in the possible sarcopenia subgroup. In contrast, the findings in the nonsarcopenia subgroup were less stable: the association remained statistically significant only in the sensitivity analysis using the −1.2 SD MCI threshold, whereas the other 4 sensitivity analyses did not retain statistical significance. These results suggest that the association observed in the nonsarcopenia subgroup should be interpreted cautiously. In the confirmed sarcopenia subgroup, no consistent association was observed across sensitivity analyses (Table S7, Supplemental Digital Content 8). Furthermore, the nonlinear association was reproduced with the reference set at the lowest HR (Fig. S2, Supplemental Digital Content 9), and the distribution of METS-IR was visualized (Fig. S3, Supplemental Digital Content 10).

In prediction analyses, we further repeated the incremental and substitutional evaluations after restricting the sample to participants within the 1st to 99th percentile of METS-IR. In the incremental value analysis, the significant improvement previously observed for AB was no longer statistically significant after excluding extreme values, indicating that the incremental value of adding METS-IR to LE-8 was not robust to outlier exclusion (Table S8, Supplemental Digital Content 11). In the substitutional value analysis, the 2-component substitution model, in which METS-IR substituted for BMI and blood glucose, showed significant results only in SVM and CB after outlier exclusion; this was directionally consistent with the main analysis, with a positive ΔAUC in SVM and a negative ΔAUC in CB, whereas the significant findings for XGB and LGBM were no longer retained. In the 3-component substitution model, in which METS-IR substituted for BMI, blood glucose, and blood lipids, significant improvements persisted in SVM, MLP, and KNN, consistent with the main analysis. In the 4-component substitution model, in which METS-IR substituted for BMI, blood glucose, blood lipids, and blood pressure, significant improvements also persisted in DT, SVM, MLP, and KNN, consistent with the main analysis. Overall, these findings suggest that the substitutional value of METS-IR was model-dependent and partly affected by extreme values, but several substitutional patterns remained consistent after outlier exclusion (Table S9, Supplemental Digital Content 12).

## 4. Discussion

To our knowledge, this study is the 1st to demonstrate, across strata of sarcopenia status, the longitudinal association between METS-IR and incident MCI, and to evaluate both the incremental and substitutional value of METS-IR based on LE-8 for predicting MCI. This focus aligns with the Lancet Commission’s evidence that targeting modifiable metabolic risks is paramount for global dementia prevention.^[27]^ We found a significant nonlinear association between METS-IR and the risk of incident MCI, with the lowest risk observed at intermediate-to-higher METS-IR levels, which has been identified in previous research.^[28]^ Our study showed that this association was statistically significant in the overall sample and in the possible sarcopenia subgroup. In the nonsarcopenia subgroup, some METS-IR quartiles were associated with a lower hazard of incident MCI, but the overall nonlinear pattern appeared less robust. The association was not supported by sufficient statistical evidence in the confirmed sarcopenia subgroup. Furthermore, we identified that adding METS-IR provided subtle improvement in prediction models based on LE-8, but METS-IR-containing reduced-feature models that omitted biochemical LE-8 components showed comparable or improved discrimination in selected algorithms. However, this substitution effect was model-dependent and requires external validation before practical use.

Prior studies have generally supported that insulin resistance is associated with cognitive decline and an increased risk of dementia. Mechanistically, impaired brain insulin signaling has been linked to disrupted energy metabolism, neuroinflammation, vascular dysfunction, and amyloid/τ-related changes.^[29]^ As Arnold et al highlighted, brain insulin resistance serves as a unifying pathological link that accelerates neurodegenerative pathways like neuroinflammation and synaptic loss.^[30]^ However, evidence is inconsistent when focusing specifically on METS-IR as a surrogate index of IR. Some studies in nondiabetic Chinese populations reported a positive association between METS-IR and MCI risk, which contrasts with our nonlinear association.^[31]^ These discrepancies may be explained by differences in study design (cross-sectional vs longitudinal follow-up), analytic framework (logistic regression vs time-to-event models), sample volume (1142 vs 4980) and inclusion/exclusion criteria (e.g., excluding baseline diabetes vs only excluding baseline memory-related disorders). Notably, there is evidence consistent with our nonlinear pattern, where compared with participants in the lowest quartile, those in the highest quartile of METS-IR had an 18% lower risk of cognitive impairment.^[28]^ A plausible explanation is reverse causation related to the prodromal phase of dementia. Longitudinal evidence indicates that weight loss can begin years to more than a decade before dementia diagnosis and may accelerate prior to clinical onset. If follow-up overlaps with this prodromal window, lower baseline BMI or lower metabolic markers may reflect ongoing preclinical neurodegeneration rather than a healthier metabolic state.^[32]^ Moreover, the association between BMI and dementia risk may differ across the life course, often positive in midlife but inverse in late life. Because the METS-IR formula incorporates BMI along with lipid and glucose information, it may capture a mixed signal in older adults that reflects both metabolic risk and body-weight/nutritional reserve.^[33]^

Previous longitudinal evidence has established an association between sarcopenia and incident MCI.^[22]^ However, in current research with sarcopenia status stratification, within confirmed sarcopenia subgroups, the association between METS-IR and MCI lacked statistical support. In sarcopenia, cognitive risk may be driven more strongly by frailty, inflammatory burden, reduced physical activity, and multimorbidity, which could attenuate the relative contribution of METS-IR within the causal pathway.^[34]^ Smaller sample size, fewer events, and a narrower distribution of metabolic indicators within sarcopenia subgroups may limit statistical power, producing nonsignificant results even if the underlying direction is similar.

Prospective evidence indicates that higher LE-8 is associated with lower dementia incidence and/or better cognitive performance.^[35,36]^ In our study, prediction models based on LE-8 demonstrated modest discriminatory performance, supporting the relevance of LE-8 for cognitive risk prediction. Furthermore, the incremental value of METS-IR beyond LE-8 was limited and algorithm-specific, and the AB-specific improvement observed in the main analysis was not retained after excluding extreme METS-IR values. METS-IR-containing reduced-feature models that omitted biochemical LE-8 components (BMI, blood glucose, blood lipids, and blood pressure) achieved higher discrimination in several algorithms. This may improve prediction accuracy while reducing the burden of routine risk screening in practice. However, because these improvements were modest, model-dependent, and only internally validated, they should be interpreted as exploratory rather than evidence of immediate practical substitution. The significant association we identified between METS-IR and MCI in the possible sarcopenia subgroup is consistent with previous findings showing a synergistic effect between METS-IR and possible sarcopenia.^[37]^

Several limitations should be acknowledged. First, the exclusion of many participants because of unavailable METS-IR components, missing baseline cognitive data, or lack of follow-up information may have introduced selection bias and limited the representativeness of the analytic sample. Second, although we adjusted for multiple demographic, lifestyle, and clinical factors, residual confounding from unmeasured or imprecisely measured factors cannot be excluded. Third, our predictive models were internally evaluated, and external validation in independent populations is required before clinical implementation. Fourth, although discrimination was evaluated across multiple machine-learning algorithms, calibration performance was generally imperfect in several models, as shown by the calibration plots and calibration metrics. In addition, extreme METS-IR values may influence both regression estimates and predictive model performance. Although we repeated the Cox and prediction analyses after restricting METS-IR to the 1st to 99th percentile, the prediction results remained algorithm-dependent, suggesting that these findings should be interpreted as exploratory rather than directly clinically actionable. Therefore, the predicted probabilities should be interpreted with caution, and these models should not be used directly for individual risk estimation without further calibration and external validation. Finally, CHARLS does not provide direct dietary structure data, and the LE-8 diet component was therefore approximated using available food-related expenditure variables, which may have introduced measurement error.

## 5. Conclusions

METS-IR showed a significant association with incident MCI in the overall sample and among participants with possible sarcopenia. In machine-learning models, the incremental value of METS-IR beyond LE-8 was small and algorithm-specific. METS-IR-containing reduced models showed modest, model-dependent discrimination after replacing selected metabolic LE-8 components, but these findings require external validation before clinical use.

## Acknowledgments

The authors extend sincere appreciation to the participants and workers of the CHARLS study for their essential contributions.

## Author contributions

**Formal analysis:** Xuzhou Zhu.

**Supervision:** Chunbo Liu.

**Writing – original draft:** Xuzhou Zhu, Chenyue Wang, Qinghua Cao, Qian Xu, Xiaoli Guo, Chunbo Liu.

**Writing – review & editing:** Xuzhou Zhu, Chenyue Wang, Qinghua Cao, Qian Xu, Xiaoli Guo, Chunbo Liu.











**Figure:**
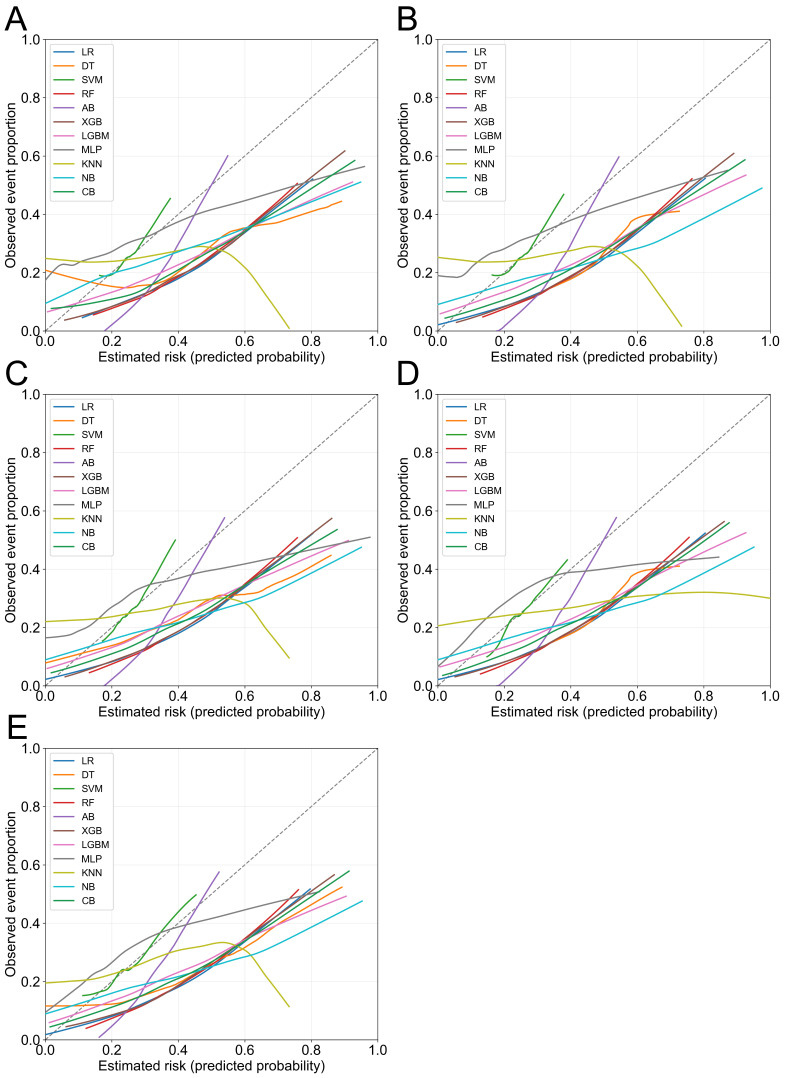






**Figure:**
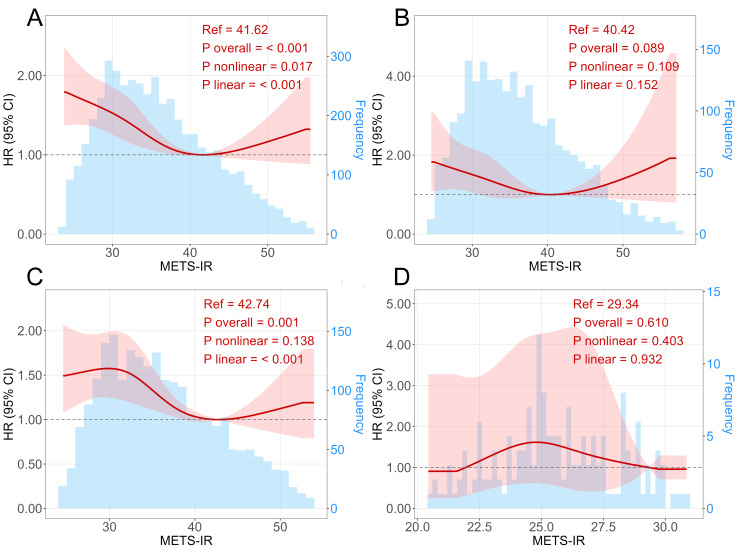


**Figure:**