# Supplementary material for: Substitutional value of METS-IR for biochemical components of life’s essential 8 in predicting incident mild cognitive impairment: A longitudinal cohort study
Source: Medicine (Baltimore). 2026 Jun 12;105(24):e49278. doi: 10.1097/MD.0000000000049278 (PMC13268502; doi:10.1097/MD.0000000000049278)
Supplement: Supplementary file 1 [file medi-105-e49278-s001.docx]

**Supplemental Table 1. Inclusion and Exclusion of Population.**

| **Steps** | Number  before | Number  excluded | Number  remaining |
| --- | --- | --- | --- |
| **Initial cohort** | 25586 | | |
| **Exclude: Age <45** | 25586 | 602 | 24984 |
| **Exclude: METS-IR unavailable** | 24984 | 16437 | 8547 |
| **Exclude: Lack of data to determine MCI at baseline** | 8547 | 1903 | 6644 |
| **Exclude: Baseline MCI** | 6644 | 1230 | 5414 |
| **Exclude: Baseline age missing** | 5414 | 29 | 5385 |
| **Exclude: Baseline age not classifiable into age groups*** | 5385 | 2 | 5383 |
| **Exclude: Memory impairment** | 5383 | 64 | 5319 |
| **Exclude: Drug for memory problem** | 5319 | 3 | 5316 |
| **Exclude: Lost to follow-up** | 5316 | 336 | 4980 |
| **Final overall population** | 4980 | | |

Age group* refers to the 5-year age bands required for defining MCI according to Aging-Associated Cognitive Decline criteria. METS-IR, metabolic score for insulin resistance; MCI, mild cognitive impairment.
