# Supplementary material for: Substitutional value of METS-IR for biochemical components of life’s essential 8 in predicting incident mild cognitive impairment: A longitudinal cohort study
Source: Medicine (Baltimore). 2026 Jun 12;105(24):e49278. doi: 10.1097/MD.0000000000049278 (PMC13268502; doi:10.1097/MD.0000000000049278)
Supplement: Supplementary file 2 [file medi-105-e49278-s002.docx]

**Supplemental Table 2. Comparison of included and excluded participants.**

| **Characteristic** | **Included analytic sample (n=4,980)** | **Included available n** | **Excluded participants (n=20,606)** | **Excluded available n** | **P value** | **SMD** |
| --- | --- | --- | --- | --- | --- | --- |
| **METS-IR** | 37.48 ± 52.62 | 4980 | 34.81 ± 8.38 | 3817 | **<0.001** | 0.071 |
| **Age (years)** | 57.91 ± 8.44 | 4980 | 58.73 ± 10.78 | 12553 | **0.042** | 0.084 |
| **Sex** |  | 4980 |  | 20598 | **<0.001** | **0.140** |
| Female | 2298 (46.1%) |  | 10946 (53.1%) |  |  |  |
| Male | 2682 (53.9%) |  | 9652 (46.9%) |  |  |  |
| **Education** |  | 4980 |  | 20562 | **<0.001** | **0.261** |
| Below Primary School | 1637 (32.9%) |  | 8640 (42.0%) |  |  |  |
| Primary School | 1304 (26.2%) |  | 5896 (28.7%) |  |  |  |
| Middle School | 1310 (26.3%) |  | 3518 (17.1%) |  |  |  |
| High school and above | 729 (14.6%) |  | 2508 (12.2%) |  |  |  |
| **Residence** |  | 4980 |  | 12683 | 0.423 | 0.013 |
| Rural village | 3841 (77.1%) |  | 9853 (77.7%) |  |  |  |
| Urban community | 1139 (22.9%) |  | 2830 (22.3%) |  |  |  |
| **Alcohol** |  | 4980 |  | 12572 | **<0.001** | **0.126** |
| None | 3131 (62.9%) |  | 8654 (68.8%) |  |  |  |
| Yes | 1849 (37.1%) |  | 3918 (31.2%) |  |  |  |
| **Body mass index** | 24.68 ± 37.01 | 4980 | 23.85 ± 35.08 | 8651 | **<0.001** | 0.023 |
| **nHDL-C (mg/dL)** | 143.90 ± 38.40 | 4980 | 140.80 ± 39.64 | 6670 | **<0.001** | 0.079 |
| **FBG (mg/dL)** | 110.14 ± 35.61 | 4980 | 109.60 ± 34.72 | 5462 | 0.903 | 0.015 |
| **HbA1c (%)** | 5.29 ± 0.81 | 4951 | 5.24 ± 0.83 | 6755 | **<0.001** | 0.050 |
| **SBP (mmHg)** | 128.39 ± 20.54 | 4937 | 130.26 ± 22.08 | 8803 | **<0.001** | 0.088 |
| **DBP (mmHg)** | 75.41 ± 12.03 | 4937 | 75.52 ± 12.38 | 8802 | 0.696 | 0.009 |
| **Diabetes mellitus diagnosed** |  | 4936 |  | 12472 | **0.013** | 0.042 |
| No | 4599 (93.2%) |  | 11747 (94.2%) |  |  |  |
| Yes | 337 (6.8%) |  | 725 (5.8%) |  |  |  |
| **Hypertension diagnosed** |  | 4960 |  | 12510 | 0.553 | 0.010 |
| No | 3657 (73.7%) |  | 9278 (74.2%) |  |  |  |
| Yes | 1303 (26.3%) |  | 3232 (25.8%) |  |  |  |
| **Stroke diagnosed** |  | 4967 |  | 12559 | **<0.001** | 0.069 |
| No | 4868 (98.0%) |  | 12172 (96.9%) |  |  |  |
| Yes | 99 (2.0%) |  | 387 (3.1%) |  |  |  |
| **Sarcopenia status** |  | 4980 |  | 20606 | **<0.001** | **0.551** |
| Confirmed sarcopenia | 181 (3.6%) |  | 403 (2.0%) |  |  |  |
| Possible sarcopenia | 2383 (47.9%) |  | 4694 (22.8%) |  |  |  |
| Non-sarcopenia | 2416 (48.5%) |  | 15509 (75.3%) |  |  |  |
| **LE-8 (score 0-100)** |  |  |  |  |  |  |
| Diet | 52.91 ± 42.24 | 4701 | 54.08 ± 43.19 | 11862 | 0.325 | 0.027 |
| Physical activity | 66.92 ± 46.34 | 2152 | 60.46 ± 48.35 | 4757 | **<0.001** | **0.136** |
| Nicotine exposure | 58.72 ± 43.69 | 4966 | 65.71 ± 41.74 | 11894 | **<0.001** | **0.164** |
| Sleep | 71.13 ± 32.02 | 4961 | 66.89 ± 34.37 | 11130 | **<0.001** | **0.128** |
| Body mass index | 87.42 ± 19.83 | 4980 | 89.25 ± 19.05 | 8651 | **<0.001** | 0.094 |
| Blood lipids | 65.30 ± 30.23 | 4980 | 67.82 ± 30.52 | 6670 | **<0.001** | 0.083 |
| Blood glucose | 91.69 ± 20.55 | 4935 | 92.02 ± 19.94 | 6694 | 0.555 | 0.017 |
| Blood pressure | 60.00 ± 36.08 | 4938 | 57.60 ± 36.91 | 8803 | **<0.001** | 0.066 |

Categorical variables are shown as n (%); continuous variables are shown as mean ± SD.

Alcohol indicates whether the individual consumed alcohol during the past year. Diabetes mellitus, hypertension and stroke status were based on doctor diagnosis. Sarcopenia status was classified in this study. Characteristics of included and excluded participants were not imputed, so the summed comorbidity counts do not always equal the total number of participants.

METS-IR, metabolic score for insulin resistance; nHDL-C, non–high-density lipoprotein cholesterol; FBG, fasting blood glucose; HbA1c, glycated hemoglobin A1c; SBP, systolic blood pressure; DBP, diastolic blood pressure; LE-8, Life’s Essential 8; SMD, standardized mean difference.
