# Supplementary material for: Substitutional value of METS-IR for biochemical components of life’s essential 8 in predicting incident mild cognitive impairment: A longitudinal cohort study
Source: Medicine (Baltimore). 2026 Jun 12;105(24):e49278. doi: 10.1097/MD.0000000000049278 (PMC13268502; doi:10.1097/MD.0000000000049278)
Supplement: Supplementary file 3 [file medi-105-e49278-s003.docx]

**Supplemental Table 3. Associations of METS-IR and Mild Cognitive Impairment Stratified by Sarcopenia Status.**

| **METS-IR**  **Quartile (range)** | **Number of**  **cases/total** | | | **Unadjusted**  **HR (95% CI)** | **Multivariate model**  **HR (95% CI)** | | **P**  **value** |
| --- | --- | --- | --- | --- | --- | --- | --- |
| **Non-sarcopenia (n=2416)** | | | | | | | |
| Q1 (7.59 - 30.72) | | 167/604 | Ref | | | Ref | - |
| Q2 (30.73 - 35.34) | | 125/604 | 0.71 (0.56, 0.89) | | | 0.69 (0.48, 0.97) | **0.036** |
| Q3 (35.35 - 41.29) | | 116/604 | 0.66 (0.52, 0.84) | | | 0.56 (0.38, 0.80) | **0.002** |
| Q4 (41.30 - 3442.56) | | 114/604 | 0.63 (0.50, 0.81) | | | 0.76 (0.53, 1.10) | 0.147 |
| **Possible sarcopenia (n=2383)** | | | | | | | |
| Q1 (15.83 - 30.47) | | 197/596 | Ref | | | Ref | - |
| Q2 (30.50 - 35.25) | | 187/596 | 0.92 (0.76, 1.13) | | | 1.01 (0.82, 1.23) | 0.951 |
| Q3 (35.27 - 41.23) | | 136/595 | 0.63 (0.51, 0.79) | | | 0.68 (0.54, 0.85) | **< 0.001** |
| Q4 (41.24 - 751.62) | | 145/596 | 0.69 (0.55, 0.85) | | | 0.71 (0.56, 0.90) | **0.005** |
| **Confirmed sarcopenia (n=** **181)** | | | | | | | |
| Q1 (19.09 - 23.99) | | 8/46 | Ref | | | Ref | - |
| Q2 (23.99 - 25.46) | | 14/45 | 1.78 (0.75, 4.26) | | | 1.66 (0.68, 4.03) | 0.276 |
| Q3 (25.54 - 27.58) | | 14/45 | 1.67 (0.70, 3.99) | | | 2.10 (0.84, 5.23) | 0.122 |
| Q4 (27.64 - 35.69) | | 7/45 | 0.91 (0.33, 2.52) | | | 0.71 (0.25, 2.02) | 0.521 |

The multivariate Cox model was adjusted for age, sex, education, physical activity, sleep, smoking, alcohol use, systolic blood pressure, diabetes, and lipid-lowering medication use.

METS-IR, metabolic score for insulin resistance; MCI, mild cognitive impairment; HR, hazard ratio; CI, confidence interval; Ref, reference.
