# Supplementary material for: Substitutional value of METS-IR for biochemical components of life’s essential 8 in predicting incident mild cognitive impairment: A longitudinal cohort study
Source: Medicine (Baltimore). 2026 Jun 12;105(24):e49278. doi: 10.1097/MD.0000000000049278 (PMC13268502; doi:10.1097/MD.0000000000049278)
Supplement: Supplementary file 4 [file medi-105-e49278-s004.docx]

**Supplemental Table 4. Incremental Value of METS-IR against LE-8.**

| **Model** | **Delta**  **AUC** | **P**  **value** |
| --- | --- | --- |
| **LR** | -0.0005 | 0.622966 |
| **DT** | -0.00975 | 0.051749 |
| **SVM** | -0.0003 | 0.522871 |
| **RF** | 0.00195 | 0.261733 |
| **AdaBoost** | **0.004868** | **< 0.05** |
| **XGBoost** | 0.003216 | 0.149898 |
| **LightGBM** | 0.007089 | 0.119173 |
| **MLP** | 0.016907 | 0.208606 |
| **KNN** | 0.000722 | 0.2177 |
| **NB** | -0.00076 | 0.512595 |
| **CatBoost** | 0.004281 | 0.193723 |

Incremental value was reported as delta AUC of LE-8 predictors plus METS-IR vs LE-8 predictors solely.

METS-IR, metabolic score for insulin resistance; LE-8, Life’s Essential 8; AUC, Area under curve; LR, Logistic Regression; DT, Decision Tree; SVM, Support Vector Machine; RF, Random Forest; AdaBoost, Adaptive Boosting; XGBoost, eXtreme Gradient Boosting; LightGBM, Light Gradient Boosting Machine; MLP, Multilayer Perceptron; KNN, k-Nearest Neighbors; NB, Naïve Bayes; CatBoost, Categorical Boosting.
