# Supplementary material for: Substitutional value of METS-IR for biochemical components of life’s essential 8 in predicting incident mild cognitive impairment: A longitudinal cohort study
Source: Medicine (Baltimore). 2026 Jun 12;105(24):e49278. doi: 10.1097/MD.0000000000049278 (PMC13268502; doi:10.1097/MD.0000000000049278)
Supplement: Supplementary file 5 [file medi-105-e49278-s005.docx]

**Supplemental Table 5. Substitutional Value of METS-IR against LE-8.**

| **Model** | **AUC**  **Baseline** | **Delta**  **AUC 1*** | **P**  **value** | **Delta**  **AUC 2*** | **P**  **value** | **Delta**  **AUC 3*** | **P**  **value** |
| --- | --- | --- | --- | --- | --- | --- | --- |
| **LR** | 0.692761 | -0.00038 | 0.723428 | 0.000179 | 0.869446 | 0.000484 | 0.690652 |
| **DT** | 0.666671 | 0.007648 | 0.114311 | ≈0 | ≈1 | **0.01534** | **< 0.001** |
| **SVM** | 0.556977 | **0.014886** | **< 0.001** | **0.024102** | **< 0.001** | **0.03893** | **< 0.01** |
| **RF** | 0.696829 | -0.00031 | 0.633652 | 0.000526 | 0.514342 | 0.002041 | 0.054651 |
| **AdaBoost** | 0.701291 | -0.00022 | 0.774758 | -5.8E-05 | 0.944977 | -0.00242 | 0.147322 |
| **XGBoost** | 0.698651 | **-0.00298** | **< 0.05** | -0.00181 | 0.297 | -0.00165 | 0.423897 |
| **LightGBM** | 0.680616 | **-0.00649** | **< 0.05** | -0.00386 | 0.330078 | -0.00787 | 0.090223 |
| **MLP** | 0.606459 | 0.017076 | 0.106703 | **0.05169** | **< 0.001** | **0.04252** | **< 0.001** |
| **KNN** | 0.52577 | 0.006989 | 0.384891 | **0.022031** | **< 0.05** | **0.04408** | **< 0.001** |
| **NB** | 0.687693 | 0.000189 | 0.861394 | 0.000228 | 0.834214 | -0.00059 | 0.613701 |
| **CatBoost** | 0.689101 | **-0.0049** | **< 0.05** | -0.00265 | 0.345871 | -0.00485 | 0.15484 |

Substitutional value was reported as delta AUC of LE-8 predictors plus METS-IR vs LE-8 predictors plus METS-IR minus LE-8 components stepwisely.

1* represents LE-8 predictors plus METS-IR minus BMI and blood glucose vs LE-8 predictors plus METS-IR.

2* represents LE-8 predictors plus METS-IR minus BMI, blood glucose and blood lipids vs LE-8 predictors plus METS-IR.

3* represents LE-8 predictors plus METS-IR minus BMI, blood glucose, blood lipids and blood pressure vs LE-8 predictors plus METS-IR.

METS-IR, metabolic score for insulin resistance; LE-8, Life’s Essential 8; AUC, Area under curve; LR, Logistic Regression; DT, Decision Tree; SVM, Support Vector Machine; RF, Random Forest; AdaBoost, Adaptive Boosting; XGBoost, eXtreme Gradient Boosting; LightGBM, Light Gradient Boosting Machine; MLP, Multilayer Perceptron; KNN, k-Nearest Neighbors; NB, Naïve Bayes; CatBoost, Categorical Boosting; BMI, Body mass index.
