# Supplementary material for: Substitutional value of METS-IR for biochemical components of life’s essential 8 in predicting incident mild cognitive impairment: A longitudinal cohort study
Source: Medicine (Baltimore). 2026 Jun 12;105(24):e49278. doi: 10.1097/MD.0000000000049278 (PMC13268502; doi:10.1097/MD.0000000000049278)
Supplement: Supplementary file 6 [file medi-105-e49278-s007.docx]

**Supplemental Table 6. Calibration performance of prediction models.**

| Model | Brier score | Calibration intercept | Calibration slope | P  value |
| --- | --- | --- | --- | --- |
| LE-8 component-score predictors | | | | |
| LR | 0.222 | -1.115 | 0.963 | **<0.001** |
| DT | 0.227 | -1.124 | 0.721 | **<0.001** |
| SVM | 0.185 | 0.005 | 1.255 | 0.601 |
| RF | 0.219 | -1.080 | 1.036 | **<0.001** |
| AB | 0.187 | -0.580 | 2.312 | **<0.001** |
| XGB | 0.220 | -1.092 | 0.949 | **<0.001** |
| LGBM | 0.215 | -0.962 | 0.591 | **<0.001** |
| MLP | 0.203 | 1.050 | 0.195 | **<0.001** |
| KNN | 0.196 | -0.007 | 0.021 | **<0.001** |
| NB | 0.208 | -0.708 | 0.261 | **<0.001** |
| CB | 0.216 | -1.029 | 0.755 | **<0.001** |
| LE-8 predictors plus METS-IR (incremental model) | | | | |
| LR | 0.223 | -1.114 | 0.898 | **<0.001** |
| DT | 0.225 | -1.118 | 0.956 | **<0.001** |
| SVM | 0.185 | 0.006 | 1.255 | 0.567 |
| RF | 0.218 | -1.074 | 1.038 | **<0.001** |
| AB | 0.187 | -0.584 | 2.360 | **<0.001** |
| XGB | 0.218 | -1.085 | 0.956 | **<0.001** |
| LGBM | 0.210 | -0.923 | 0.609 | **<0.001** |
| MLP | 0.196 | 0.818 | 0.200 | **<0.001** |
| KNN | 0.196 | -0.007 | 0.020 | **<0.001** |
| NB | 0.250 | -1.405 | 0.253 | **<0.001** |
| CB | 0.213 | -1.005 | 0.775 | **<0.001** |
| 2-component substitution model | | | | |
| LR | 0.223 | -1.115 | 0.926 | **<0.001** |
| DT | 0.227 | -1.119 | 0.638 | **<0.001** |
| SVM | 0.184 | 0.002 | 1.282 | **0.008** |
| RF | 0.218 | -1.075 | 1.033 | **<0.001** |
| AB | 0.186 | -0.569 | 2.302 | **<0.001** |
| XGB | 0.219 | -1.087 | 0.937 | **<0.001** |
| LGBM | 0.213 | -0.932 | 0.585 | **<0.001** |
| MLP | 0.189 | 0.434 | 0.337 | **<0.001** |
| KNN | 0.195 | 0.005 | 0.040 | **<0.001** |
| NB | 0.249 | -1.398 | 0.255 | **<0.001** |
| CB | 0.215 | -1.011 | 0.740 | **<0.001** |
| 3-component substitution model | | | | |
| LR | 0.223 | -1.115 | 0.932 | **<0.001** |
| DT | 0.225 | -1.118 | 0.956 | **<0.001** |
| SVM | 0.183 | -0.003 | 1.211 | **<0.001** |
| RF | 0.218 | -1.076 | 1.047 | **<0.001** |
| AB | 0.186 | -0.572 | 2.310 | **<0.001** |
| XGB | 0.219 | -1.087 | 0.939 | **<0.001** |
| LGBM | 0.213 | -0.946 | 0.595 | **<0.001** |
| MLP | 0.182 | 0.374 | 0.552 | **<0.001** |
| KNN | 0.216 | -0.037 | 0.025 | **<0.001** |
| NB | 0.249 | -1.399 | 0.255 | **<0.001** |
| CB | 0.215 | -1.023 | 0.759 | **<0.001** |
| 4-component substitution model | | | | |
| LR | 0.223 | -1.114 | 0.930 | **<0.001** |
| DT | 0.225 | -1.123 | 0.750 | **<0.001** |
| SVM | 0.182 | -0.009 | 1.086 | 0.177 |
| RF | 0.219 | -1.081 | 1.036 | **<0.001** |
| AB | 0.180 | -0.465 | 2.022 | **<0.001** |
| XGB | 0.219 | -1.091 | 0.937 | **<0.001** |
| LGBM | 0.216 | -0.967 | 0.586 | **<0.001** |
| MLP | 0.182 | 0.393 | 0.504 | **<0.001** |
| KNN | 0.191 | -0.021 | 0.063 | **<0.001** |
| NB | 0.249 | -1.400 | 0.254 | **<0.001** |
| CB | 0.216 | -1.029 | 0.739 | **<0.001** |

Calibration metrics were calculated using out-of-fold predicted probabilities. The Hosmer-Lemeshow test used deciles of predicted risk to obtain p-values. p-values <0.05 indicate evidence of lack of fit.

METS-IR replaces the LE-8 body mass index and blood glucose components in the 2-component substitution model. METS-IR replaces the LE-8 components for body mass index, blood glucose, and blood lipids in the 3-component substitution model. METS-IR replaces the LE-8 components of body mass index, blood glucose, blood lipids, and blood pressure in the 4-component substitution model.

LR, Logistic Regression; DT, Decision Tree; SVM, Support Vector Machine; RF, Random Forest; AB, AdaBoost; XGB, XGBoost; LGBM, LightGBM; MLP, Multilayer Perceptron; KNN, k-Nearest Neighbors; NB, Naive Bayes; CB, CatBoost; LE-8, Life’s Essential 8; METS-IR, metabolic score for insulin resistance.
