# Supplementary material for: Substitutional value of METS-IR for biochemical components of life’s essential 8 in predicting incident mild cognitive impairment: A longitudinal cohort study
Source: Medicine (Baltimore). 2026 Jun 12;105(24):e49278. doi: 10.1097/MD.0000000000049278 (PMC13268502; doi:10.1097/MD.0000000000049278)
Supplement: Supplementary file 7 [file medi-105-e49278-s008.docx]

**Supplemental Table 7. Sensitivity Analyses.**

| **Overall** | **Q1** | **Q2** | **Q3** | **Q4** | **P for trend** |
| --- | --- | --- | --- | --- | --- |
| **Sensitivity analysis 1** | Ref | 0.94 (0.80, 1.10) | 0.71 (0.59, 0.84) | 0.77 (0.64, 0.92) | **< 0.001** |
| **Sensitivity analysis 2** | Ref | 1.00 (0.83, 1.20) | 0.70 (0.57, 0.86) | 0.73 (0.59, 0.91) | **< 0.001** |
| **Sensitivity analysis 3** | Ref | 0.94 (0.79, 1.11) | 0.65 (0.53, 0.78) | 0.72 (0.59, 0.87) | **< 0.001** |
| **Sensitivity analysis 4** | Ref | 0.83 (0.66, 1.05) | 0.60 (0.46, 0.77) | 0.66 (0.51, 0.86) | **< 0.001** |
| **Sensitivity analysis 5** | Ref | 0.91 (0.77, 1.09) | 0.66 (0.54, 0.80) | 0.72 (0.58, 0.90) | **< 0.001** |
| **Non-sarcopenia** | **Q1** | **Q2** | **Q3** | **Q4** | **P for trend** |
| **Sensitivity analysis 1** | Ref | 0.77 (0.56, 1.05) | 0.63 (0.45, 0.88) | 0.76 (0.55, 1.07) | 0.062 |
| **Sensitivity analysis 2** | Ref | 0.71 (0.48, 1.04) | 0.54 (0.36, 0.82) | 0.64 (0.42, 0.98) | **0.015** |
| **Sensitivity analysis 3** | Ref | 0.69 (0.48, 0.97) | 0.56 (0.38, 0.80) | 0.76 (0.53, 1.10) | 0.072 |
| **Sensitivity analysis 4** | Ref | 0.68 (0.47, 0.98) | 0.58 (0.40, 0.85) | 0.71 (0.49, 1.05) | 0.052 |
| **Sensitivity analysis 5** | Ref | 0.67 (0.47, 0.96) | 0.53 (0.36, 0.76) | 0.77 (0.54, 1.11) | 0.073 |
| **Possible sarcopenia** | **Q1** | **Q2** | **Q3** | **Q4** | **P for trend** |
| **Sensitivity analysis 1** | Ref | 1.01 (0.83, 1.23) | 0.75 (0.61, 0.92) | 0.80 (0.64, 1.00) | **0.006** |
| **Sensitivity analysis 2** | Ref | 1.11 (0.89, 1.39) | 0.76 (0.60, 0.97) | 0.82 (0.63, 1.05) | **0.016** |
| **Sensitivity analysis 3** | Ref | 1.01 (0.82, 1.23) | 0.68 (0.54, 0.85) | 0.71 (0.56, 0.90) | **< 0.001** |
| **Sensitivity analysis 4** | Ref | 0.94 (0.69, 1.28) | 0.62 (0.44, 0.88) | 0.66 (0.46, 0.94) | **0.004** |
| **Sensitivity analysis 5** | Ref | 1.01 (0.82, 1.25) | 0.71 (0.56, 0.90) | 0.71 (0.54, 0.94) | **0.006** |
| **Confirmed sarcopenia** | **Q1** | **Q2** | **Q3** | **Q4** | **P for trend** |
| **Sensitivity analysis 1** | Ref | 2.43 (1.03, 5.69) | 2.35 (0.96, 5.78) | 1.79 (0.72, 4.41) | 0.318 |
| **Sensitivity analysis 2** | Ref | 1.48 (0.57, 3.89) | 1.33 (0.44, 4.00) | 0.43 (0.12, 1.52) | 0.207 |
| **Sensitivity analysis 3** | Ref | 1.66 (0.68, 4.03) | 2.10 (0.84, 5.23) | 0.71 (0.25, 2.02) | 0.681 |
| **Sensitivity analysis 4** | Ref | 1.71 (0.34, 8.52) | 3.90 (0.62, 24.49) | 0.62 (0.12, 3.32) | 0.545 |
| **Sensitivity analysis 5** | Ref | 0.81 (0.34, 1.96) | 1.23 (0.48, 3.13) | 0.43 (0.15, 1.20) | 0.248 |

The multivariate Cox model was adjusted for age, sex, education, physical activity, sleep, smoking, alcohol use, systolic blood pressure, diabetes, and lipid-lowering medication use. Major results reported as hazard ratio (95% confidence interval).

Sensitivity analysis 1, mild cognitive impairment (MCI) using alternative thresholds of −0.8 SD below the age-specific mean; Sensitivity analysis 2, mild cognitive impairment using alternative thresholds of −1.2 SD below the age-specific mean; Sensitivity analysis 3, a stricter baseline exclusion strategy based on a −1.2 SD cutoff while retaining the main −1 SD definition for incident MCI; Sensitivity analysis 4, repeating Cox models without MICE imputation (complete-case adjustment); Sensitivity analysis 5, excluding METS-IR outliers by including only participants within the 1st to 99th percentile of METS-IR.

Ref, reference.
