# Supplementary material for: Substitutional value of METS-IR for biochemical components of life’s essential 8 in predicting incident mild cognitive impairment: A longitudinal cohort study
Source: Medicine (Baltimore). 2026 Jun 12;105(24):e49278. doi: 10.1097/MD.0000000000049278 (PMC13268502; doi:10.1097/MD.0000000000049278)
Supplement: Supplementary file 8 [file medi-105-e49278-s011.docx]

**Supplemental Table 8. Incremental Value of METS-IR against LE-8:
sensitivity analysis restricted to the 1st-99th percentile of METS-IR.**

| **Model** | **Delta**  **AUC** | **P**  **value** |
| --- | --- | --- |
| **LR** | **0.004336** | **< 0.05** |
| **DT** | **-0.012241** | **< 0.05** |
| **SVM** | **0.002438** | **< 0.001** |
| **RF** | 0.002555 | 0.159973 |
| **AdaBoost** | 0.003499 | 0.148049 |
| **XGBoost** | 0.001653 | 0.497105 |
| **LightGBM** | 0.002811 | 0.516662 |
| **MLP** | -0.006157 | 0.587279 |
| **KNN** | 0.001089 | 0.450839 |
| **NB** | **0.002296** | **< 0.05** |
| **CatBoost** | **0.004336** | **< 0.05** |

Incremental value was reported as delta AUC of LE-8 predictors plus METS-IR vs LE-8 predictors solely, after restricting the analysis to the 1st-99th percentile of METS-IR.

METS-IR, metabolic score for insulin resistance; LE-8, Life’s Essential 8; AUC, Area under curve; LR, Logistic Regression; DT, Decision Tree; SVM, Support Vector Machine; RF, Random Forest; AdaBoost, Adaptive Boosting; XGBoost, eXtreme Gradient Boosting; LightGBM, Light Gradient Boosting Machine; MLP, Multilayer Perceptron; KNN, k-Nearest Neighbors; NB, Naïve Bayes; CatBoost, Categorical Boosting.
