# Supplementary material for: Substitutional value of METS-IR for biochemical components of life’s essential 8 in predicting incident mild cognitive impairment: A longitudinal cohort study
Source: Medicine (Baltimore). 2026 Jun 12;105(24):e49278. doi: 10.1097/MD.0000000000049278 (PMC13268502; doi:10.1097/MD.0000000000049278)
Supplement: Supplementary file 9 [file medi-105-e49278-s012.docx]

**Supplemental Table 9. Substitutional Value of METS-IR against LE-8:
sensitivity analysis restricted to the 1st-99th percentile of METS-IR.**

| **Model** | **AUC**  **Baseline** | **Delta**  **AUC 1*** | **P**  **value** | **Delta**  **AUC 2*** | **P**  **value** | **Delta**  **AUC 3*** | **P**  **value** |
| --- | --- | --- | --- | --- | --- | --- | --- |
| **LR** | 0.69512 | -0.001537 | 0.214061 | -0.0015 | 0.260067 | -0.001106 | 0.410119 |
| **DT** | 0.664736 | 0.007131 | 0.155429 | 0.000657 | 0.494576 | **0.011331** | **< 0.05** |
| **SVM** | 0.545675 | **0.012302** | **< 0.001** | **0.020161** | **< 0.001** | **0.039841** | **< 0.01** |
| **RF** | 0.694425 | -0.000425 | 0.468054 | -0.000427 | 0.57446 | 0.000145 | 0.886713 |
| **AdaBoost** | 0.698447 | 0.000751 | 0.211815 | **0.001622** | **< 0.05** | **-0.002943** | **< 0.05** |
| **XGBoost** | 0.693733 | -0.00152 | 0.244659 | 0.001069 | 0.468834 | -0.001426 | 0.47466 |
| **LightGBM** | 0.671317 | 0.000931 | 0.768534 | 0.002889 | 0.448398 | -0.001619 | 0.715903 |
| **MLP** | 0.582945 | **0.036849** | **< 0.01** | **0.029417** | **< 0.01** | **0.076325** | **< 0.001** |
| **KNN** | 0.519955 | 0.003391 | 0.675301 | **0.024245** | **< 0.05** | **0.033613** | **< 0.01** |
| **NB** | 0.687287 | 0.00088 | 0.289243 | 0.001189 | 0.157189 | 0.000655 | 0.46915 |
| **CatBoost** | 0.687272 | **-0.007316** | **< 0.01** | -0.003343 | 0.238014 | -0.005567 | 0.114835 |

Substitutional value was reported as delta AUC of LE-8 predictors plus METS-IR vs LE-8 predictors plus METS-IR minus LE-8 components stepwisely, after restricting the analysis to the 1st-99th percentile of METS-IR.

1* represents LE-8 predictors plus METS-IR minus BMI and blood glucose vs LE-8 predictors plus METS-IR.

2* represents LE-8 predictors plus METS-IR minus BMI, blood glucose and blood lipids vs LE-8 predictors plus METS-IR.

3* represents LE-8 predictors plus METS-IR minus BMI, blood glucose, blood lipids and blood pressure vs LE-8 predictors plus METS-IR.

METS-IR, metabolic score for insulin resistance; LE-8, Life’s Essential 8; AUC, Area under curve; LR, Logistic Regression; DT, Decision Tree; SVM, Support Vector Machine; RF, Random Forest; AdaBoost, Adaptive Boosting; XGBoost, eXtreme Gradient Boosting; LightGBM, Light Gradient Boosting Machine; MLP, Multilayer Perceptron; KNN, k-Nearest Neighbors; NB, Naïve Bayes; CatBoost, Categorical Boosting; BMI, Body mass index.
